# Membrane associated collagen XIII promotes cancer metastasis and enhances anoikis resistance

**DOI:** 10.1186/s13058-018-1030-y

**Published:** 2018-10-01

**Authors:** Hui Zhang, Tricia Fredericks, Gaofeng Xiong, Yifei Qi, Piotr G. Rychahou, Jia-Da Li, Taina Pihlajaniemi, Wei Xu, Ren Xu

**Affiliations:** 1 Department of Laboratory Medicine, The First Hospital of Jilin University, Changchun, 130021 Jilin Province China; 2 UK Markey Cancer Center, University of Kentucky, Lexington, KY 40536 USA; 3 Department of Pharmacology and Nutritional Sciences, University of Kentucky, Lexington, KY 40536 USA; 4 Division of Gynecologic Oncology, Department of Obstetrics and Gynecology, University of Kentucky, Lexington, KY 40504 USA; 5 Department of Surgery, College of Medicine, University of Kentucky, Lexington, KY 40504 USA; 6 Center for Medical Genetics, School of Life Sciences, Central South University, Changsha, 410078 Hunan Province China; 7 Center for Cell-Matrix Research and Biocenter Oulu, Faculty of Biochemistry and Molecular Medicine, University of Oulu, 90014 Oulu, Finland

**Keywords:** Extracellular matrix, Membrane-associated collagen, Stemness, Cell anoikis, Metastasis

## Abstract

**Background:**

Increased collagen expression and deposition are associated with cancer progression and poor prognosis in breast cancer patients. However, function and regulation of membrane-associated collagen in breast cancer have not been determined. Collagen XIII is a type II transmembrane protein within the collagen superfamily. Experiments in tissue culture and knockout mouse models show that collagen XIII is involved in cell adhesion and differentiation of certain cell types. In the present study, we determined roles of collagen XIII in breast cancer progression and metastasis.

**Methods:**

We analyzed the association of collagen XIII expression with breast cancer development and metastasis using published gene expression profiles generated from human breast cancer tissues. Utilizing gain- and loss- of function approaches and 3D culture assays, we investigated roles of collagen XIII in regulating invasive tumor growth. Using the tumorsphere/mammosphere formation assay and the detachment cell culture assay, we determined whether collagen XIII enhances cancer cell stemness and induces anoikis resistance. We also inhibited collagen XIII signaling with β1 integrin function-blocking antibody. Finally, using the lung colonization assay and the orthotopic mammary tumor model, we investigated roles of collagen XIII in regulating breast cancer colonization and metastasis. Cox proportional hazard (log-rank) test, two-sided Student’s t-test (two groups) and one-way ANOVA (three or more groups) analyses were used in this study.

**Results:**

Collagen XIII expression is significantly higher in human breast cancer tissue compared with normal mammary gland. Increased collagen XIII mRNA levels in breast cancer tissue correlated with short distant recurrence free survival. We showed that collagen XIII expression promoted invasive tumor growth in 3D culture, enhanced cancer cell stemness, and induced anoikis resistance. Collagen XIII expression induced β1 integrin activation. Blocking β1 integrin activation significantly reduced collagen XIII-induced invasion and mammosphere formation. Importantly, silencing collagen XIII in MDA-MB-231 cells reduced lung colonization and metastasis.

**Conclusions:**

Our results demonstrate a novel function of collagen XIII in promoting cancer metastasis, cell invasion, and anoikis resistance.

**Electronic supplementary material:**

The online version of this article (10.1186/s13058-018-1030-y) contains supplementary material, which is available to authorized users.

## Background

Despite the recent progress in hormone and targeted therapy, breast cancer still remains the second cause of cancer-related death in women. Most of breast cancer death is due to metastasis [1]. Therefore, it is important to determine how the metastasis process is regulated and to identify potential targets for repressing cancer metastasis. Breast cancers can be classified into luminal (A and B), Her2, and basal-like/triple-negative breast cancer (TNBC) based on the expression status of estrogen receptor, progesterone receptor, and Her2/neu [2].

Increased collagen expression and deposition are associated with cancer progression and poor prognosis in breast cancer patients [3]. For instance, type I collagen has been identified as a prognosis marker and is associated with cancer recurrence in human breast cancer patients [4]. Collagen VI knockout mice have reduced primary tumor formation and growth [5]. Importantly, the transgenic mice with increased collagen deposition in mammary tissue have a three-fold increase in tumor formation. These mice also have three times more lung metastases [6]. In addition, aligned collagen fibers can facilitate cell migration and metastasis [7, 8]. These results suggest that increased collagen deposition can transform cells into a malignant phenotype and promote cancer metastasis [9]. Dense breast is a risk factor for breast cancer [10], and the increased breast density is due in part to increased deposition of collagen proteins [11]. In addition, the extracellular matrix (ECM) surrounding a breast tumor is more dense or stiff from increased collagen deposition and crosslink [12]. These researches indicate that increased collagen expression and deposition promotes breast cancer development and progression by enhancing tumor growth and invasion.

The collagen family can be divided into several groups based on the protein structure and localization [13]. One group of collagen, including collagen XIII, collagen XXIII, and collagen XXV, is a type II transmembrane protein [14–17]. Collagen XIII protein is 90 to 100 kDa and folds in an opposite fashion to the fibrillar collagens. It has a membrane spanning region near the NC1 domain, which results in a large extracellular region with a short intracellular portion [17]. The function of collagen XIII at the molecular level has largely remained unclear; however, it is thought to have a role in cell-cell and cell-matrix interactions [18]. The extracellular domain binds integrin [19], and can be cleaved from the cell resulting in possible paracrine activity in the cellular microenvironment. When the ectodomain is shed, the pericellular surrounding is less supportive of cell adhesion, migration and proliferation [20]. The function of the short intracellular portion after cleavage is unclear but has been shown to feedback and increase collagen XIII production [21, 22]. Collagen XIII mRNA has been shown to be expressed in higher levels in epithelial tumors, such as tumors from the colon, cervix, bladder, endometrium and ovary [23, 24]. Increased collagen XIII protein expression is also detected in invasive foci of bladder cancer [24, 25]. However, the function of collagen XIII in breast cancer progression has not been determined.

Integrin is one of the cell membrane receptors that mediate the cell-collagen interaction [26, 27]. Binding of integrin to collagen induces activation of downstream signaling, and subsequently modulates cell proliferation, differentiation, apoptosis and cell migration [28–31]. Expression of β1 integrin is required for the epithelial integrity and plays a crucial role in the proliferation of mammary epithelial cells [32]. Treatment with a β1 integrin functional blocking antibody can reverse breast cancer cells back to the normal phenotype in 3D culture [33]. It has been shown in a mouse mammary tumor model that disruption of β1 integrin function inhibits tumor development at the initial stages of mammary tumor formation [34]. These results suggest that β1 integrin is a key mediator of collagen-induced cancer development and progression.

In this study, we showed that expression of collagen XIII is higher in breast cancer tissue compared with normal mammary gland, and that the increased mRNA level of collagen XIII in cancer tissue is associated with poor prognosis and cancer metastasis. We also demonstrate that collagen XIII expression enhanced cancer stemness and invasive tumor growth through β1 integrin. Importantly, silencing collagen XIII in breast cancer cells significantly reduced cancer metastasis. These results identified a novel function of membrane associated collagen in breast cancer progression.

## Methods

### Cell lines and culture conditions

MDA-MB-231 (ATCC) were maintained in DMEM/F12 with 10% FBS and 1% Pen/Strep. BT549 (ATCC) cells were maintained in RPMI-1640 with 10% FBS and 1% Pen/Strep. MCF-10A cells were kind gifts from Michael W. Kilgore, University of Kentucky, Lexington, KY. MCF10A cells were cultured as previously described [35]. Hs-578 T cells (ATCC) were kept as previously described [36]. S1 and T4–2 cells are kind gifts from Dr. Mina J Bissell, and they were cultured as previously described [37]. All the cells were tested for mycoplasma contamination every two months.

### Antibodies and reagents

Anti-flag M2 (Sigma, F1804, 1:1000 for WB, 1:500 for IF). Anti-human β1 integrins,active (Millipore, MAB2079Z, 1:500 for IF). Anti-human Collagen XIII α1 (R&D systems, AF6346, 1:200 for WB). Anti-smad2/3 (BD Transduction Laboratories, 610842, 1:1000 for WB). Anti-p-smad2 (cell signaling, 130D4, 1:1000 for WB). Anti-tubulin (Cell Signaling, 2148, 1:5000). Anti-Integrin β1 subunit (AIIB2) (DSHB, 528306, 80 μg/ml for block assay). Anti-rat IgG1 (Santa Cruz Biotechology, sc-3882, 1:1000). Anti-PARP (Cell Signaling, 9542, 1:1000). Bovine Collagen Solution, Type I (Advanced BioMatrix, 5005).

Dual-luciferase reporter assay system (Promega, E1960). Click-It EdU Alexa Fluor 488 Imaging kit (Invitrogen, C10337). Growth Factor Reduced BD Matrigel™ (BD Biosciences, 354230). Annexin V (Thermo, A13201). LentiCRISPR v2 (Addgene, 52961), pCDH-EF1-MCS-T2A-Puro (System Biosciences, CD520A-1), p3TP-lux (Addgene, 306281), pGL4.10 (Addgene, 66128).

### Plasmid construction

Mouse Collagen XIII (NM_007731.3) were amplified from constructs pCMV-SPORT6-COL13a1 (Transomic, BC034164), and cloned into pCDH-EF1-MCS-T2A-Puro (System Biosciences, CD520A-1) with the primers of

Forward: 5’ AATTGAATTCGCCACCATGGTGGCGGAGCGCACCCGC 3′;

Reverse: 5’ACTGGCGGCCGCCTTATCGTCGTCATCCTTGTAATCCTGCCCTCCAGGCCTGCTTCT3’.

Human Collagen XIII (NM_001130103.1) was amplified from an expression construct described by Dennis et al. [38], and cloned into FLAG-tagged pCDH-EF1-MCS-T2A-Puro with the primers of

Forward: 5’AATTGCTAGCGCCACCATGGTAGCGGAGCGCACCCAC 3′;

Reverse: 5’ACTGGAATTCCTTGTTCCAGCAGCCTTGGAC 3′.

### 3D culture

Three-dimensional (3D) IrECM on-top culture was performed as previous described [39]. Briefly, Growth Factor Reduced BD Matrigel™ was plated on the bottom of the cell culture dish. MDA-MB-231 and MCF10A cells were seeded on the top of the matrigel layer, and additional medium containing 10% Matrigel was added on the top.

### CRISPR-Cas9 deletion of collagen XIII in MDA-MB-231 and T4–2 cells

CRISPR-Cas9 plasmid for collagen XIII (NM_001130103.1) deletion was constructed with gDNA primers: 5’ CACCGCAGCTCGGCCGTCCGAAAGT 3′ (Forward) and 5’AAACACTTTCGGACGGCCGAGCTGC 3′ (Reverse). MDA-MB-231 cells were infected with the lentivirus containing the CRISPR-Cas9 construct, and monoclones were selected and verified by genomic DNA sequencing and western blot. The collagen XIII-knockout luciferase-expressing or GFP-expressing MDA-MB-231 cells were pooled together for the mouse experiments.

### Western blot and luciferase reporter assay

Protein samples were harvested by using 2% SDS in PBS with protease inhibitor cocktail and NaF (2.5 mM), NaVO4 (2 mM). SDS gel electrophoresis, immunoblot and a LI-COR Odyssey Infrared Imaging System were employed for detecting the target protein as previously described [39]. The Image Studio Lite software was used for quantification.

The Dual-luciferase reporter assay was performed as previously described [40]. Briefly, MDA-MB-231cells or MCF10A cells were seeded into a 24-well plate at the density of 0.1 × 10^6^/well. 24 h later the cells will reach 80% confluence. Then 0.5 μg p3TP-lux and 0.025 μg renilla plasmids were transfected into the cells using Fugene HD Transfection Reagent. The cells were starved before 5 ng/ml TGF-β was added. The relative luciferase activity was defined as firefly luciferase activity normalized by renilla luciferase activity. The final results were normalized by the relative luciferase activity of the control vector pGL4.10.

### Transwell invasion and single cell migration assay

The Transwell invasion assay was performed as previously described [41]. As for the single cell migration assay, MCF-10A and MDA-MB-231 cells were seeded into a 4 chamber glass bottom dish (Invitro Scientific, D35C4–20-1-N) at the density of 2000 cell/cm^2^. About 4 h after seeding, the single cell migration was monitored by Nikon BioStation (Nikon, IMQ) every 10 min for 10 h [41]. In some experiments, the cells were pre-incubated with β1 integrin blocking antibody AIIB2 with the final concentration of 80 μg/ml for 30 min, and then the Transwell invasion and single cell migration assay were performed in the presence of the blocking antibody.

### Mammosphere/Tumorsphere assay

Cells were seeded in poly-HEMA (12 mg/ml in 95% ethanol) pre-coat regular plastic culture dish [42] and cultured in tumorsphere medium [DMEM/F12 medium supplemented with B27 (1:50), EGF (20 ng/ml), bFGF (20 ng/ml), insulin (5 μg/ml), hydrocortisone (0.5 ng/ml), Gentamicin (10 μg/ml)] for 5 days without moving or disturbing the plates. The phase images of mammosphere/tumorsphere were taken by Nikon eclipse 80i microscope. Mammosphere or tumorspheres forming efficiency (%) was calculated as follows: (Number of mammosphere or tumorspheres per well / number of cells seeded per well) × 100.

### Flow cytometry analysis apoptosis

Poly-HEMA (12 mg/ml in 95% ethanol) pre-coated dishes were used for detachment cell culture [42]. MCF-10A and MDA-MB-231 cells were cultured in regular media with 0.5% methyl cellulose in suspension at a density of 30,000 cells per cm^2^ [42]. Cells were cultured in suspended condition for 24 h. And then were collected for annexin V analysis as manufacturer’s instructions.

### Immunofluorescence staining

For immunofluorescence staining, cells were cultured in a chamber slide (Nalge Nunc International, 154526). The cells were fixed with Methanol/Acetone (1:1) or formalin and permeabilized with 0.5% Triton X-100. The slides were blocked by 10% goat serum at room temperature for 60 min, and incubated with the primary antibodies (anti-flag/anti-active-β1 integrin/anti-caspase 3) at 4 °C overnight. The slides were incubated with secondary antibodies at room temperature for 60 min. Images were taken with Nikon Eclipse 80i fluorescence microscope and Nikon eclipse Ti2 confocal microscope.

### Cell proliferation assay

Cell proliferation was performed per the instructions of the Click-It EdU Alexa Fluor 488 Imaging kit and assessed by quantification of the proportion of cells with EdU-positive staining. The number of nuclei positive for EdU was counted and divided by the total number of nuclei (DAPI).

### Xenograft experiment and in vivo colonization experiments

For the xenograft experiment, 6-week old female SCID mice were randomly grouped and injected with 2 × 10^6^ control or collagen XIII-silenced MDA-MB-231-luc-D3H2LN cells at 4th mammary fat pad. Tumors were measured with a caliper every other day. Tumor volume (mm^3^) was estimated using the formula [volume = π × (width)^2^ × (length) / 6]. Twenty five days after tumor cell implantation, the primary tumors are removed by surgery. To detect lung metastasis, bioluminescent images were taken at 3 weeks after primary tumor removal with in vivo imaging system (IVIS).

For lung colonization experiment, 6-week old female SCID mice were randomly grouped and injected with 0.5 × 10^6^ (in 200 μl PBS) control or collagen XIII-silenced MDA-MB-231-luc-D3H2LN cells via tail vein. To detect lung metastasis, bioluminescent images were taken once a week begining 4 weeks after the injection of cancer cells with IVIS. At the experimental endpoint, lung tissues were harvested and fixed with 4% PFA for paraffin-embedded section. H&E staining was performed in lung tissue sections, and images were taken by a Nikon microscope. Metastasized tumors in the lung were quantified by counting three sections per lung sample. For the intracardiac inoculation experiment, 6-week old female nude mice were randomly grouped. 0.2 × 10^6^ (in 100 μl PBS) control, collagen XIII-silenced MDA-MB-231-luc-D3H2LN cells or collagen XIII-silenced MDA-MB-231-GFP cells were injected into left cardiac ventricle. Bioluminescent images were taken once a week to detect lung and bone metastasis. Illumatool was used to detect GFP labeled cells metastasis.

### Statistical analysis

To address the clinical relevance of increased collagen XIII expression, we assessed the association between mRNA levels of Col13A1 and recurrence or distant recurrence free survival using the published microarray dataset generated from 3554 human breast cancer tissue samples [43] (2014 version). Patients were equally grouped into low and high Col13A1 expression based on the mRNA levels. Significant differences in recurrence or distant recurrence survival time were assessed with the Cox proportional hazard (log-rank) test.

All experiments were conducted by three independent experiments. Data were reported as mean ± s.e.m.. Student’s *t*-test (two groups) or one-way ANOVA (three or more groups) were used to determine the significant differences between means. Statistical analysis was performed with Graph Pad Prism 5 and IBM SPSS Statistics 22. *p* < 0.05 represents statistical significance and *p* < 0.01 represents sufficiently statistical significance. All reported *p* values were from two-sided tests.

## Results

### Collagen XIII expression is increased during breast cancer development

**Fig. 1:**
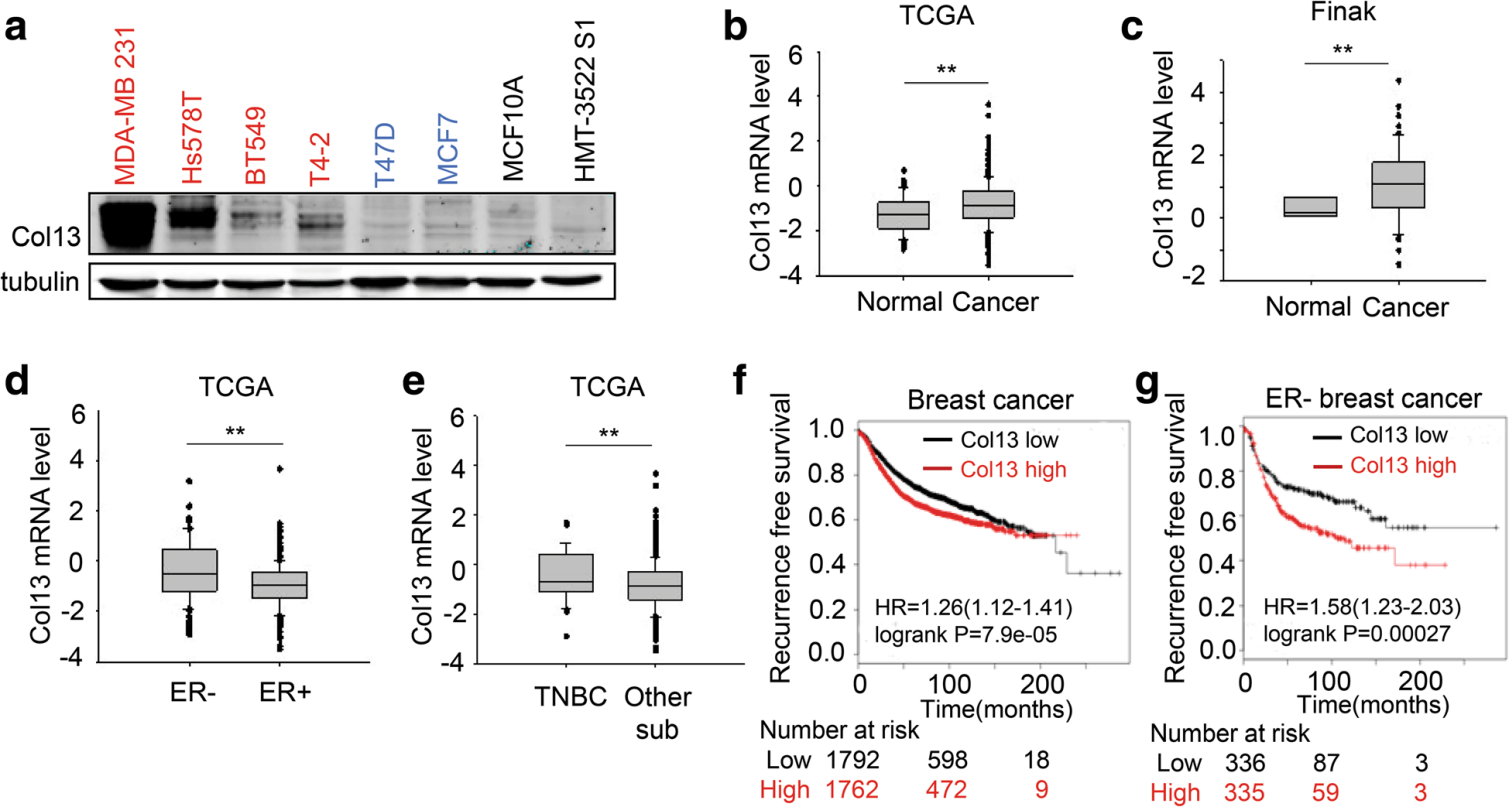
Collagen XIII expression is increased during breast cancer development. **a** Western blot analysis of Collagen XIII (Col13) in the malignant and non-malignant mammary epithelial cell lines in vitro. Red stands for triple negative breast cancer cell lines, blue stands for luminal type breast cancer cell lines, black stands for non-malignant mammary epithelial cell lines. **b** Col13 mRNA expression in human breast cancer and normal mammary tissues in the TCGA dataset. *n* = 593 ** *p <* 0.01. **c** Col13 mRNA expression in human breast cancer and normal mammary tissues in the Finak dataset. *n* = 59 ** *p* *<* 0.01. **d** Col13 mRNA levels in ER negative (*n* = 91) and ER positive (*n* = 270) subgroups of breast cancer patients; ** *p <* 0.01. **e** Col13 mRNA expression in triple negative breast cancer tissues (*n* = 48) and other subtypes (*n* = 473); results are presented as the mean ± s.e.m.; ** *p* *<* 0.01. **f** Kaplan-Meier analysis of recurrence free survival of breast cancer patients grouped into high and low expression levels of Col13. The patients were equally divided into two groups based on the mRNA levels of Col13 in breast cancer tissues, *n* = 3554; *** *p <* 0.001. **g** Kaplan-Meier analysis of recurrence free survival of estrogen receptor negative patients grouped into high and low expression levels of Col13. *n* = 671; *** *p* *<* 0.001

To determine whether collagen XIII expression is induced during breast cancer development, we analyzed collagen XIII protein levels in a panel of non-malignant and malignant mammary epithelial cells. Triple negative breast cancer cell line MDA-MB-231, Hs578T, BT549, and T4–2 expressed higher levels of collagen XIII protein than luminal type breast cancer cell lines and non-malignant mammary epithelial cell lines (Fig. 1a). By analyzing TCGA and Finak datasets (www.oncomine.com), we found that collagen XIII mRNA levels were significantly increased in human breast cancer tissue compared to normal mammary gland tissue (Fig. 1b, c). Collagen XIII expression in ER negative breast cancer was much higher than the expression in ER positive breast cancer (Fig. 1d). Consistence with cancer cell line data, we also found that triple negative breast cancer tissue had higher level of collagen XIII expression compared with other subtypes (Fig. 1e).

Next, we asked whether collagen XIII expression is associated with clinical outcome in human breast cancer patients. Breast cancer patients were divided into two groups based on collagen XIII mRNA levels (low and high). Kaplan-Meier log rank analysis showed that patients whose tumors had high collagen XIII expression levels had a significantly shorter overall survival period (Fig. 1f). Moreover, the association of collagen XIII expression with poor clinical outcome is stronger in ER negative breast cancer (Fig. 1g). Although collagen XIII mRNA levels are lower in ER positive breast cancer compared to ER negative cancer, increased collagen XIII expression in ER positive breast cancer is still associated with poor clinical outcome (Additional file 1: Figure S1). These results indicate that breast cancer development and progression are accompanied by increased expression of collagen XIII.

### Collagen XIII promotes invasive 3D malignant phenotypes in MDA-MB 231 cells.

**Fig. 2:**
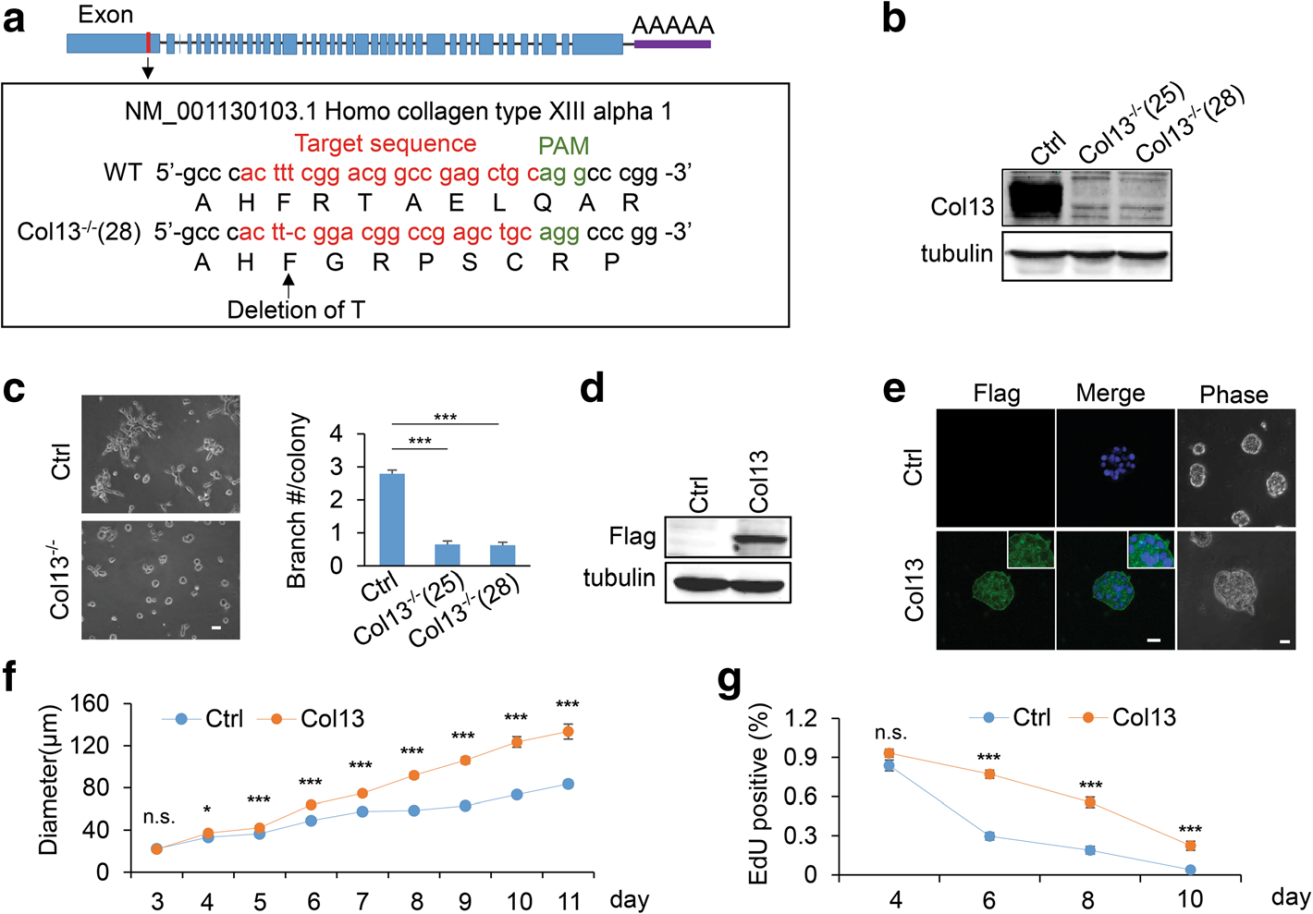
Collagen XIII promotes invasive 3D malignant phenotypes. **a** Schematic representation of human *Col13A1* gene. Blue block represents exons. The area of the exon corresponding to the target region for CRISPR/Cas9 based gene editing is highlighted in red. The genomic sequencing results of the wildtype and mutant clones are presented in the box. A deletion was detected in Col13 knockout clone (Col13^−/−^ (28)). **b** Western blot was performed to confirm Col13 knockout in Col13^−/−^ (25) and Col13^−/−^ (28) clones compared with control MDA-MB-231 cells. **c** Representative phase microscopy images of phenotype of MDA-MB-231 control and Col13^−/−^ MDA-MB-231 cells under 3D matrix culture (left). Bar graph quantifying the invasive branches in MDA-MB-231 control and two Col13^−/−^ clones (right). Results are presented as the mean ± s.e.m.; *n* = 100; *** *p* < 0.001. Scale bar: 50 μm. **d** Western blot analysis of flag-tagged Col13 and tubulin in cell lysate of control and Col13-expressing MCF-10A cells. **e** Fluorescence and phase images of control and col13-expression MCF-10A cells in 3D culture. Scale bars: 20 μm. **f** Line chart showing quantification of the diameter of control and Col13-expression MCF-10A cells at different time points in 3D culture. Data are presented as the mean ± s.e.m.; *n* = 60; * *p <* 0.05, *** *p* *<* 0.001; n.s., no significance. **g** Quantitative analysis of the cell proliferation of control and Col13-expression MCF-10A by EdU staining. Line chart representing the ratio of EdU positive to total cells. (day4, *n* = 20; day6, *n* = 20; day8, *n* = 20; day10, *n* = 20); *** *p* *<* 0.001; n.s., no significance

Increased expression of collagen XIII has been detected in several types of cancer [24, 25]. However, function of collagen XIII in cancer progression is not clear. Since collagen XIII protein is highly expressed in MDA-MB-231 cells but non-detectable in MCF-10A cells, these cell lines were used for loss- and gain-of function experiments, respectively, to define roles of collagen XIII in breast cancer progression. Collagen XIII expression was silenced in MDA-MB-231 cell line using CRISPR technology (Fig. 2a, b and Additional file 2: Figure S2). 3D culture models have been widely used to examine the malignant mammary tissue morphogenesis [44, 45]. Invasive branching structure in 3D culture is associated with cancer invasion and aggressive cancer phenotypes [36, 41]. We showed that silencing collagen XIII in MDA-MB-231 significantly reduced the number of invasive branches and inhibited invasive growth in 3D culture (Fig. 2c). For the gain-of function experiments, MCF10A cells were infected with lentivirus containing the collagen XIII expression construct. The majority of exogenous collagen XIII was detected on cell surface (Fig. 2d, 2e, and Additional file 3: Figure S3a). We found that collagen XIII expression increased 3D colony size in MCF10A cells (Fig. 2f). The ratio of EdU positive cells was significantly higher in the collagen XIII expression group compared with the control group (Fig. 2g, Additional file 3: Figure S3b). A reduction of activated caspase 3 staining was also detected in collagen XIII-expressing cells (Additional file 3: Figure S3c).

**Fig. 3:**
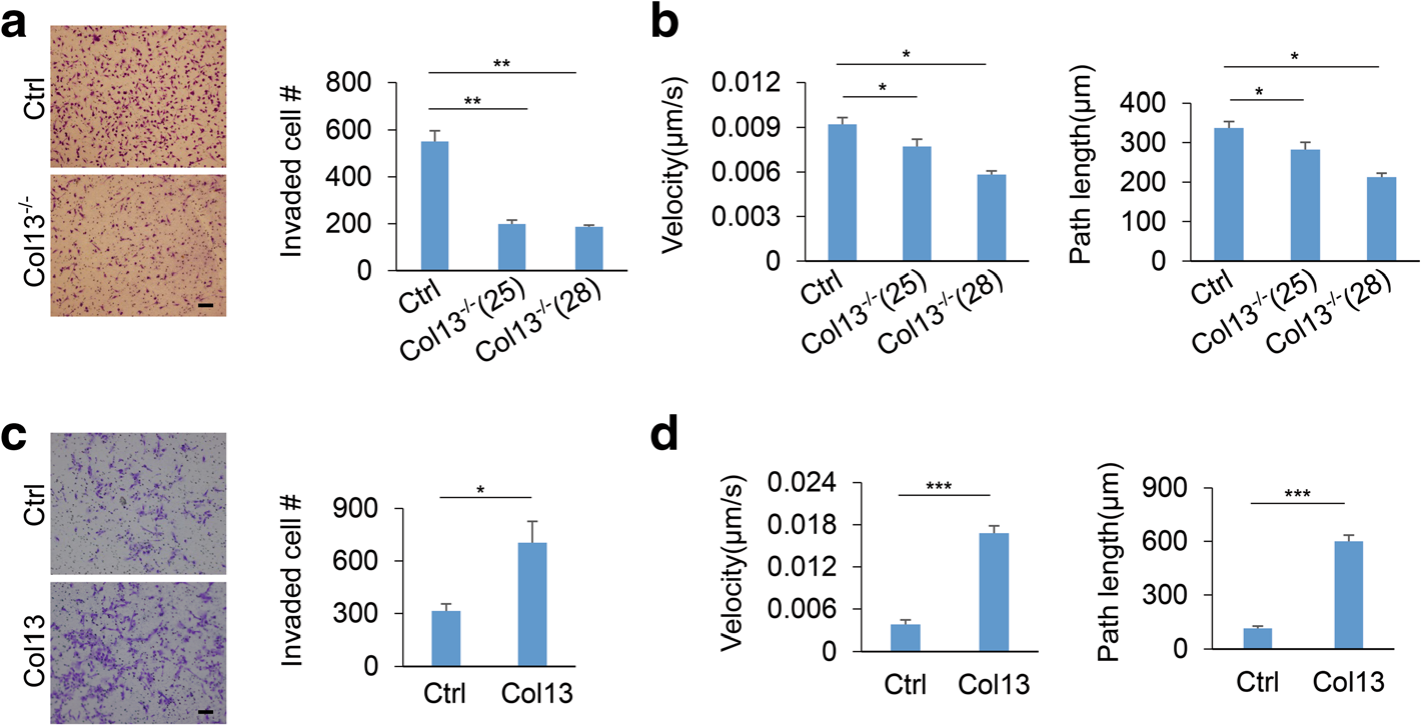
Collagen XIII enhances cancer cell migration and invasion. **a** Phase images (left) and quantification data (right) showing invasion of control and Col13^−/−^ MDA-MB-231 cells in the Transwell analysis. Data are presented as the mean ± s.e.m.; *n* = 3; ** *p <* 0.01. Scale bar: 100 **μ**m. **b** Quantification of velocity and path length of control and Col13^−/−^ MDA-MB-231 cells in the single cell migration analysis (Control, *n* = 22; clone25, *n* = 30; clone 28, *n* = 65); * *p <* 0.05. **c** Phase images (left) and quantification data (right) showing the invasion of control and Col13-expressing MCF-10A cells in Transwell experiments; n = 3; * *p* *<* 0.05. Scale bar: 100 **μ**m. **d** Quantification of velocity and path length of control and Col13-expressing MCF-10A cells in the single cell migration analysis; *n* = 25; *** *p* *<* 0.001

Invasive growth of breast cancer in 3D culture depends on cancer cell invasion and migration [46, 47]. Thus, we asked whether collagen XIII regulates invasion and migration of mammary epithelial cells. Silencing collagen XIII significantly reduced invasion of MDA-MB-231 cells in Transwell experiments (Fig. 3a). Using single cell tracking analysis, we showed that knockout of collagen XIII reduced the velocity and the distance of cell migration in MDA-MB-231 cells (Fig. 3b). In contrast, MCF-10A cells with collagen XIII overexpression were more invasive than control cells (Fig. 3c). Collagen XIII expression also significantly increased the cell migration velocity and distance in MCF-10A (Fig. 3d, and Additional file 4: Figure S4a, b). These results indicate that increased expression of collagen XIII in MDA-MB 231 cells promotes malignant phenotypes in 3D culture by enhancing cancer cell migration and invasion. Collagen XIII is expressed at the intermediate level in malignant T4–2 cells (Fig.1a), and we performed both loss- and gain-of-function experiments using this cell line. Silencing collagen XIII reduced T4–2 invasion, while overexpression of collagen XIII did not significantly enhance cell invasion in the Transwell experiments (Additional file 5: Figure S5). Therefore, the moderate expression of collagen XIII may be sufficient to promote cancer invasion. To determine whether collagen XIII regulates cell invasion in a cell-autonomous fashion, we performed co-culture experiments by mixing GFP-labeled collagen XIII-silenced MDA-MB-231 cells with non-labeled wild type MDA-MB-231 cells. We found that wild type MDA-MB-231 cells could not rescue cell invasion in the collagen XIII-silenced cells (Additional file 6: Figure S6). These results suggest that collagen XIII promotes cancer cell invasion through the receptor on the same cells.

### Collagen XIII expression enhances cancer cell stemness.

**Fig. 4:**
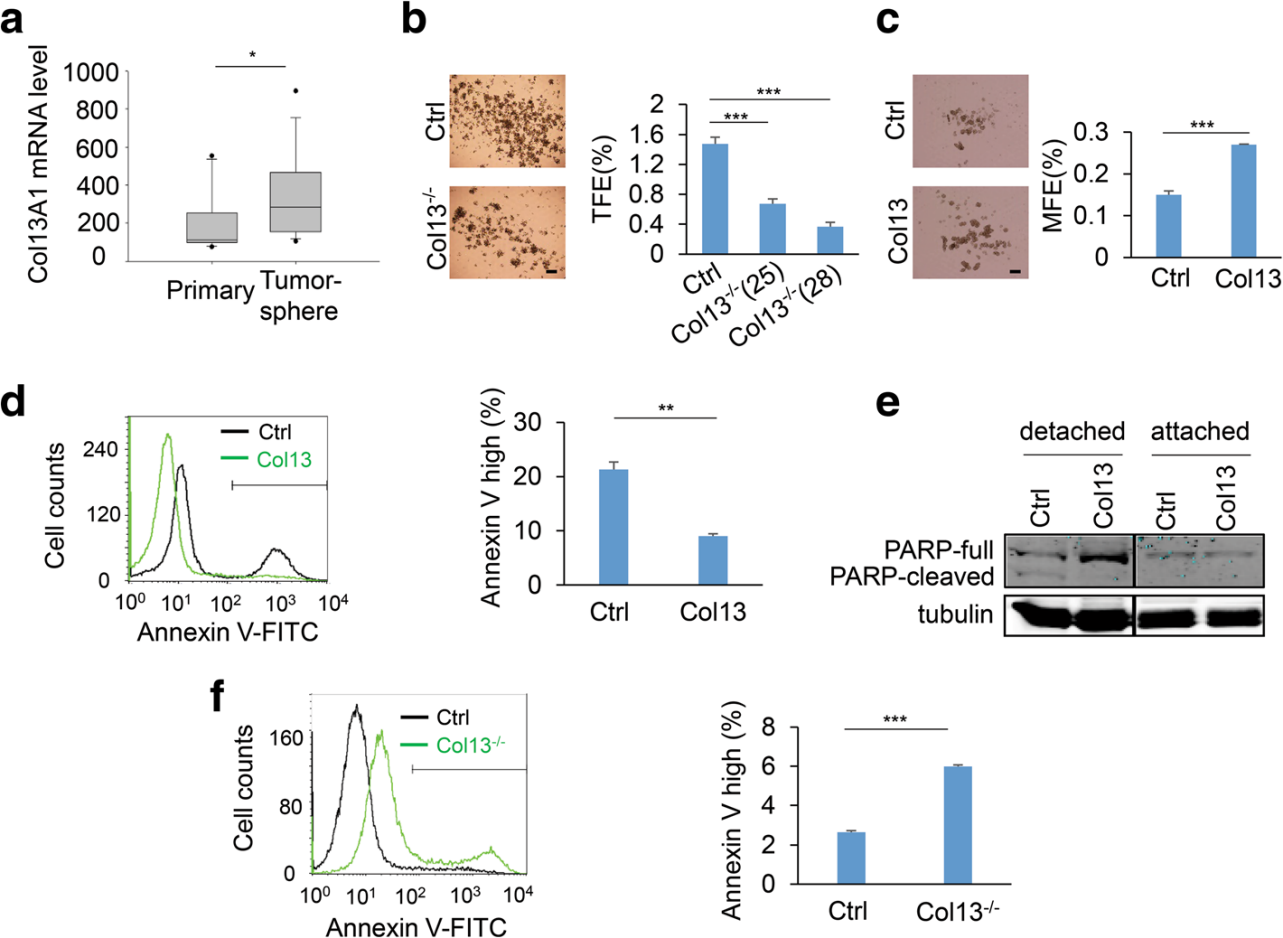
Collagen XIII expression enhances cancer cell stemness. **a** Quantification of mRNA levels of Col13 in primary tumors (*n* = 11) and tumorsphere (*n* = 15). Data are presented as the mean ± s.e.m.; * *p* < 0.05. **b** Phase images and quantification data showed tumorshpere formation in control and Col13^−/−^ MDA-MB-231 cells; *n* = 5; *** *p* *<* 0.001. Scale bar: 200 μm. **c** Phase images and quantification data showed mammosphere formation in control and Col13-expressing MCF-10A cells; n = 6; *** *p* *<* 0.001. Scale bar: 200 μm. **d**. Cell detachment induced apoptosis was quantified in control and Col13-expressing cells. Cells were cultured with 0.5% methyl cellulose in suspension for 24 h to induce anoikis before the analysis. Cell surface phosphatidylserine was analyzed by annexin-V and followed by flow cytometry analysis. Early apoptosis was quantified with bar graph; *n* = 3; ** *p* *<* 0.01. **e** Western blot analyzing PARP-cleavage in control and Col13-expressing MCF-10A cells. Cells were treated as described in d before the analysis. **f** Cell detachment induced apoptosis was quantified in control and Col13^−/−^ MDA-MB-231 as described in d. Data are presented as the mean ± s.e.m.; *n* = 3; *** *p* *<* 0.001

Tumor-initiating cells are the driver of cancer relapses and metastasis [48, 49]. The tumorsphere assay has been used to enrich tumor-initiating cells and to study their colony formation activity [50, 51]. By analyzing the published microarray dataset [52], we found that collagen XIII expression was upregulated in tumor spheroids compared to the corresponding primary tumors (Fig. 4a). To determine whether collagen XIII contributes to tumorsphere formation, we cultured control, collagen XIII-silenced MDA-MB-231 and T4–2 cells in non-adhesive plates. We found that silencing collagen XIII significantly reduced tumorsphere formation in both MDA-MB-231 and T4–2 cells (Fig. 4b and Additional file 7: Figure S7a). In contrast, expression of collagen XIII enhanced mammosphere formation in MCF10A cells (Fig. 4c). Interestingly, overexpression of collagen XIII in T4–2 had little effect on tumorsphere formation, suggesting that the moderate expression of collagen XIII is sufficient to enhance cancer cell stemness (Additional file 7: Figure S7b). Next, we performed another tumorsphere formation experiments by mixing GFP-labeled collagen XIII-silenced MDA-MB-231 cells with non-labeled wild type MDA-MB-231 cells. Wild type MDA-MB-231 cells did not increase the tumorsphere formation efficiency in GFP-labeled collagen XIII-silenced cells (Additional file 8: Figure S8). These results suggest that collagen XIII enhances cancer cell stemness in a cell-autonomous manner.

Tumor initiating cells are more resistant to detachment-induced anoikis, which is crucial for the cancer cell survival during cancer metastasis [53]. To determine whether collagen XIII promotes anoikis resistance, control, collagen XIII-silenced MDA-MB-231, and collagen-expressing MCF10A cells were cultured in methyl cellulose. Cell anoikis was assessed by analyzing PARP cleavage and cell surface-expressed phosphatidylserine with FITC-labeled annexin-V. We found that collagen XIII expression reduced detachment-induced cell surface-expressed phosphatidylserine and PARP cleavage in MCF-10A cells (Fig. 4d, e), while silencing collagen XIII increased annexin-V staining in MDA-MB-231 cells (Fig. 4f). These results suggest that collagen XIII promotes breast cancer progression by enhancing cancer cell stemness and its associated anoikis resistance.

### Collagen XIII enhances tumorsphere formation and TGF-β signaling through β1 integrin

**Fig. 5:**
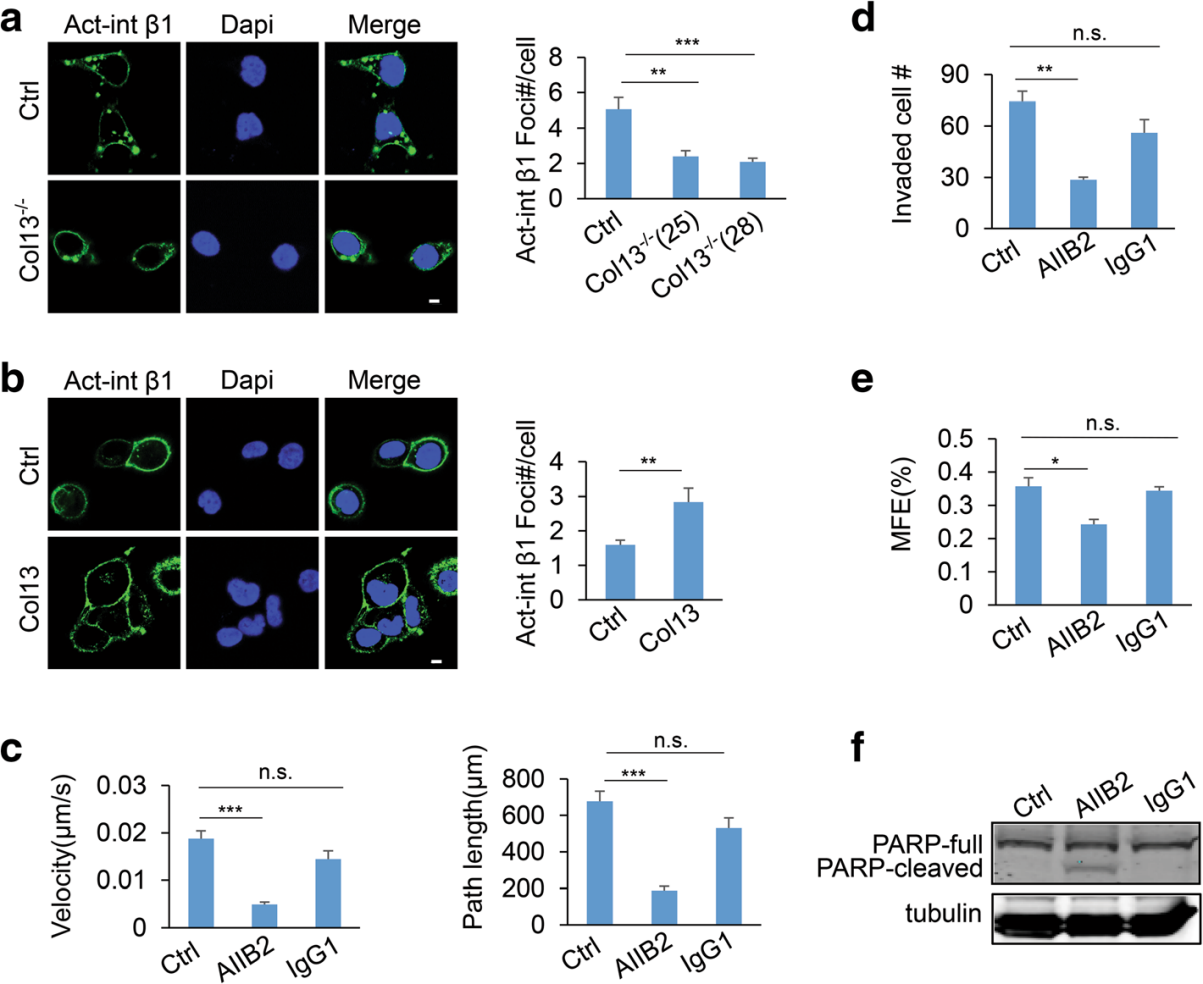
Collagen XIII enhances cancer cell invasion and stemness through β1 integrin. **a** Immune fluorescence imaging analysis of β1 integrin activation in control and Col13^−/−^ MDA-MB-231 cells (left). Bar graph quantifying the foci number of activated β1 integrin staining on cell membrane in control and Col13^−/−^ MDA-MB-231 cells (right). Data are represented as the mean ± s.e.m.; n = 10 (fields); ** *p* *<* 0.01, *** *p* *<* 0.001. Scale bar: 5 μm. **b** Immune fluorescence imaging analyzed β1 integrin activation in control and Col13-expressing MCF-10A cells (left). Bar graph quantifying the foci number of activated β1 integrin staining on cell membrane in control and Col13-expressing MCF-10A cells (right); *n* = 10 (fields); ** *p* *<* 0.01. Scale bar: 5 μm. **c** The velocity (left) and path length (right) of single cell migration were quantified in Col13-expressing MCF-10A cells in the presence of β1 integrin functional blocking antibody AIIB2 or control IgG; *n* = 27; *** *p* *<* 0.001; n.s., no significance. **d** Bar graph quantifying invasion of Col13-expressing MCF-10A cells in the presence of AIIB2 or IgG; n = 3; ** *p* *<* 0.01. **e** Bar graph quantifying the mammosphere forming efficiency of Col13-expressing MCF-10A cells in the presence of AIIB2 or IgG; *n* = 3; * *p* *<* 0.05. **f** Western blot data showing that AIIB2 treatment enhanced PARP-cleavage in Col13-expressing MCF-10A cells

To understand how collagen XIII regulates cancer progression, we determined whether collagen XIII induces β1 integrin activation. Monoclonal antibody HUTS-4 is specific against active human β1 integrin [54]. Activated β1 integrin was analyzed by immune fluorescence staining with HUTS-4 in control, collagen XIII-silenced or collagen XIII-expressing cells. Quantified data showed that silencing collagen XIII reduced the activated β1 integrin foci in MDA-MB-231 cells (Fig. 5a). In contrast, expression of collagen XIII induced β1 integrin activation in MCF-10A cells (Fig. 5b). AIIB2 is an antibody that blocks β1 integrin activity [33]. To determine whether β1 integrin activation is crucial for collagen XIII-induced cell function, the collagen XIII-expressing MCF10A cells were treated with the control IgG or AIIB2 antibody in the single cell migration, invasion, and mammosphere formation experiments. We showed that the AIIB2 treatment blocked collagen XIII-induced cell migration (Fig. 5c), invasion (Fig. 5d), and mammosphere formation in MCF10A cells (Fig. 5e), while IgG treatment had little effect. In addition, inhibition of β1 integrin activation enhanced PARP cleavage in collagen-expressing cell (Fig. 5f). These results indicate that collagen XIII enhances cell invasion and mammosphere formation at least partially through the β1 integrin pathway.

**Fig. 6:**
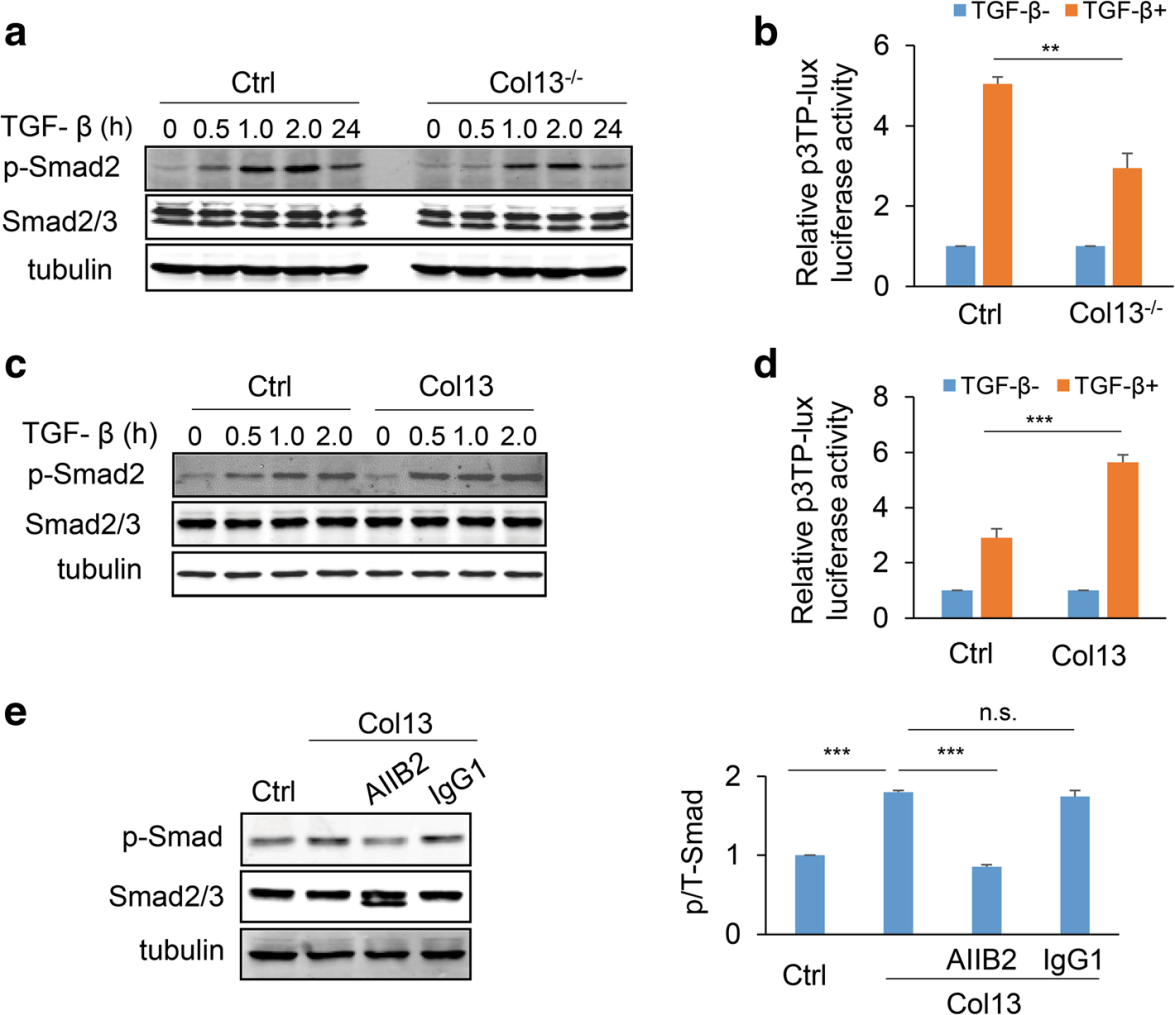
Collagen XIII enhances activation of the TGF-β pathway. **a** Western blot analyzed TGF-β-induced Smad phosphorylation in control and Col13^−/−^ MDA-MB-231 cells. **b** Bar graph quantifying TGF-β-induced TPA response elements (TREs)-driven luciferase activity in control and Col13^−/−^ MDA-MB-231; n = 3; ** *p* *<* 0.01. **c** Western blot analyzed TGF-β-induced Smad phosphorylation in control and Col13-expressing MCF-10A cells. **d** Bar graph showing TGF-β-induced TRE-driven luciferase activity in control and Col13-expressing MCF-10A cells; *n* = 4, *** *p* *<* 0.001. **e** AIIB2 treatment reduced the level of TGF-β-induced Smad2/3 phosphorylation in Col13-expressing MCF-10A cells. Western bolt images (left) and quantification (right); *n* = 3; *** *p* <0.001; n.s., no significance

Aberrant activation of the TGF-β pathway promotes cancer progression by enhancing cancer cell stemness and invasion [55–57]. It has been shown that β1 integrin contributes to the activation of the TGF-β pathway [58, 59]. We asked whether collagen XIII enhances the TGF-β signaling through β1 integrin. Control and collagen XIII knock-out MDA-MB-231 clones were treated with TGF-β1. The cell lysates at various time points were analyzed by western blot to determine the ratio of phosphorylated SMAD2/3 compared to total SMAD. The TGF-β-induced SMAD2/3 phosphorylation was decreased in the collagen XIII knockout clones compared to control MDA-MB-231 cells (Fig. 6a). Consistent with western blot data, the TGF-β-induced TPA response elements (TREs)-driven luciferase activities were significantly reduced in collagen XIII-silenced cells (Fig. 6b). These experiments were repeated in the MCF-10A cells. Results showed that the TGF-β-induced SMAD2/3 phosphorylation and reporter activities were enhanced by exogenous collagen XIII expression (Fig. 6c, d). Importantly, AIIB2 treatment significantly reduced the collagen XIII-enhanced SMAD2/3 phosphorylation (Fig. 6e). Therefore, collagen XIII expression may enhance the TGF-β signaling through β1 integrin which subsequently promotes cancer progression.

### Silencing collagen XIII inhibits breast cancer metastasis in mice

**Fig. 7:**
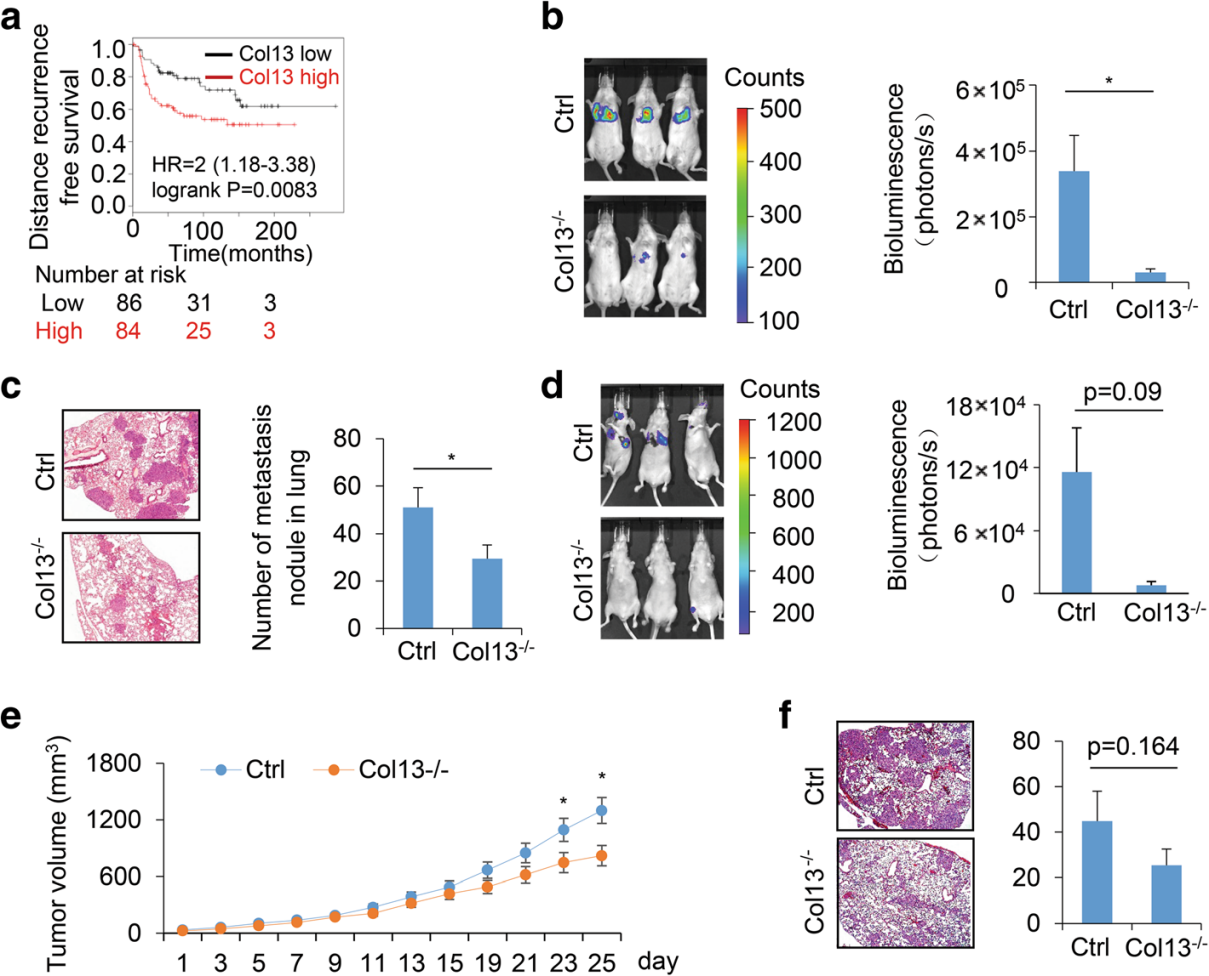
Collagen XIII promotes cancer metastasis in xenograft models. **a** Kaplan-Meier analysis of distant recurrence free survival of ER negative breast cancer patients; the patients were equally divided into high and low expression levels of collagen XIII. *n* = 170. **p <0.01. **b** IVIS images (left) and quantification (right) of tail vain lung metastasis in control and Col13^−/−^ 231-luc-D3H2LN cells injected mice. Data are presented as the mean ± s.e.m.; *n* = 5, * *p* *<* 0.05. **c** H&E staining (left) and quantification (right) of lung metastasis nodules in control and Col13^−/−^ 231-luc-D3H2LN cells injected mice; *n* = 4; * *p* *<* 0.05. **d** IVIS images (left) and quantification (right) showed over all metastasis of control and Col13^−/−^ 231-luc-D3H2LN cells via intracardiac inoculation. Data are presented as the mean ± s.e.m.; *n* = 3, *p* = 0.09. **e** Tumor growth curve of control and Col13^−/−^ 231-luc-D3H2LN implanted mice. On day 23 and 25 showed statistical significance; *n* = 6; * *p* *<* 0.05. **f** H&E staining (left) and quantification (right) of lung metastasis nodules in mice 3 weeks after primary tumor removal; *n* = 5; *p* = 0.164

We showed that expression of collagen XIII was associated with short distant recurrence free survival in patients with ER negative (Fig. 7a) and ER positive breast cancer (Additional file 9: Figure S9), suggesting that collagen XIII contributes to cancer metastasis. To determine whether collagen XIII expression promotes cancer cell colonization at distant organs, we silenced collagen XIII expression in MDA-MB-231-luc-D3H2LN cells and pooled multiple clones together (Additional file 10: Figure S10). Control and collagen XIII-silenced cells were injected into the tail veins of SCID mice. Lung colonization of the cancer cells was monitored by IVIS imaging. We showed that the mice injected with control cells developed lung metastasis within 5 weeks, while silencing collagen XIII significantly reduced the lung metastasis (Fig. 7b). Haemotoxylin and Eosin (H&E) staining further confirmed that silencing collagen XIII inhibited the lung colonization of cancer cells in SCID mice (Fig. 7c). Intracardiac inoculation of MDA-MB-231 cells has been used as a model to investigate breast cancer bone metastasis. Using this model, we also found that silencing collagen XIII reduced colonization of MDA-MB-231-luc-D3H2LN cells in nude mice (Fig. 7d). We further analyzed cancer cell colonization in bone using the GFP-labeled MDA-MB-231 cells. Interestingly, bone metastasis was detected in all four mice in the collagen-silenced group, while only three mice had bone metastasis in the control group (Additional file 11: Figure S11). Thus, function of collagen XIII in breast cancer bone metastasis remains for further clarification.

Next we defined roles of collagen XIII in primary tumor growth and cancer metastasis using the MDA-MB-231-luc-D3H2LN orthotopic mammary tumor model [60]. The orthotopic mammary tumor model is a physiologically relevant model to study cancer metastasis. It reproduces the entire metastatic process, including tumor cell dissemination from the mammary fat pad, followed by colonization and outgrowth at distant organs. The same amount of control and collagen XIII-silenced cancer cells were injected into the mammary fat pads of 6-week-old female SCID mice, and tumor growth was monitored twice per week. Then the primary tumors were removed, and mice were maintained for another month before analyzing the metastases in the lung. We found that silencing collagen XIII slightly reduced tumor growth (Fig. 7e). We also observed a reduction of lung metastases in collagen XIII-silenced group (Fig. 7f). Therefore, increased collagen XIII expression may promote cancer colonization and metastasis by enhancing cancer cell invasion and stemness.

## Discussion

Roles of interstitial collagen and BM collagen in mammary tumor development have been determined [61–63]. However, function of membrane-associated collagens in breast cancer is not well studied. We identified increased collagen XIII expression in breast cancer tissue, especially in triple negative breast cancers. Using the orthotopic mammary tumor model and lung colonization assay, we showed for the first time that collagen XIII expression is required for breast cancer cell metastasis. These results identified a novel function of membrane associated collagen in cancer progression.

Stromal cells, such as cancer-associated fibroblasts, are considered the major source of ECM protein in cancer tissue. It has been shown that collagen XIII is highly expressed in fibroblasts and localizes in the focal adhesion [24]. Interestingly, we found that collagen XIII is expressed in TNBC cell lines and tissues. The metastatic MDA-MB-231 cell line contains the highest level of collagen XIII compared to non-metastatic or non-malignant cell lines. A recent study shows that collagen XIII is expressed in the invasive bladder cancer cell line and the infiltrative bladder cancer tissue. Collagen XIII enhances cancer cell invasion in these cell lines [25]. We further show that the increased expression of collagen XIII promotes cancer cell migration and invasion through β1 integrin. Collagen I, collagen IV, laminin, and fibronectin are also produced by cancer cells and deposited in cancer tissue [36, 64–66]. These results indicate that cancer cells produce a significant amount of ECM proteins. Importantly, we demonstrate that collagen XIII is crucial for cancer cell stemness and metastasis, which provide additional insights about the cancer cell produced ECM.

Results from lung colonization experiments suggest that collagen XIII expression is crucial for cancer cell survival in circulation and colonization at distant organs. It has been shown that tumor initiating cells are the driver of cancer metastasis and initiate the colonization at distal sites [67, 68]. Silencing collagen XIII reduced tumorsphere formation in breast cancer cells, suggesting that the collagen XIII expression enhances cancer cell stemness. Cancer cells also need to acquire anoikis resistance to survive in circulation during cancer metastasis. We found that collagen XIII induces anoikis resistance in mammary epithelial cells. These results suggest that collagen XIII derived from cancer cells promotes cancer metastasis by enhancing cancer cell stemness and by inducing anoikis resistance.

Collagen XIII expression is detected in the invasion front of bladder cancer [24, 25]. Consistent with these results we show that collagen XIII is required for the invasive growth of MDA-MB-231 cells in 3D culture. Therefore, membrane protein collagen XIII may promote cancer cell metastasis at multiple stages, including dissemination from the primary tumor and colonization at the distant organs. Interestingly, collagen XIII is also involved in the inflammatory process and regulation of the immune system. It has been identified as a favorable prognostic factor in B-cell lymphoma [69]. In addition, mice expressing a mutant collagen XIII develop clonal mature B cell lineage lymphomas [70]. These results suggest that function of collagen XIII in the development of solid tumors and lymphoma may be different.

We found that collagen XIII expression induced the activation of β1 integrin. Inhibition of β1 integrin activation blocks collagen XIII-induced tumorsphere formation and TGF-β signaling, suggesting that β1 integrin is a crucial downstream target of collagen XIII in promoting cancer progression. It has been shown that integrin α1β1 mediates CHO cell spreading on collagen XIII [19]. The solid phase assay confirms the binding of europium-labeled αI domains to the collagen XIII. Our co-culture experiments suggest that collagen XIII binds to the integrin on the same cell and enhance cell invasion and tumorsphere formation in the cell-autonomous manner. Discoidin domain receptors (DDRs) are a family membrane proteins that bind to collagen separated from the integrin-β1 pathway [71, 72]. DDRs are tyrosine kinase receptors that are activated when bound to collagen, subsequently regulating cell proliferation, differentiation, survival, and migration [73]. Therefore, it is important to investigate if collagen XIII also regulates DDR activation in the future.

## Conclusions

In summary, this study identified a novel function of collagen XIII in breast cancer metastasis. We demonstrate that collagen XIII enhances breast cancer invasive growth and anoikis resistance through β1 integrin. These findings provide insights in the roles of membrane associated collagen in cancer progression, and suggest that targeting collagen XIII is a potential strategy for suppressing breast cancer progression.

## Additional files

Additional file 1: **Figure S1.** Kaplan-Meier analysis of recurrence free survival in ER positive breast cancer patients; the patients were equally divided into two groups based on the mRNA level of Col13 in breast cancer tissue. *n* = 1802. **p < 0.01. (PDF 2061 kb)

Additional file 2: **Figure S2.** Genomic DNA sequencing results of wild type and Col13 knockout MDA-MB-231 clone 28. There is a T deleted in the Col13 knockout MDA-MB-231 cells. (PDF 3096 kb)

Additional file 3: **Figure S3.** Immunofluorescence staining analyzes collagen XIII expression, EdU labeling, and caspase 3 activation. **a** 2D culture fluorescent microscopy images of MCF-10A control and Col13 overexpression cells. Flag (green), dapi (blue) and merged images. Scale bar: 20 μm. **b** EdU staining fluorescent microscopy images of MCF-10A control and Col13 overexpression cells. Representative image was on day6 of 3D culture. EdU (green), dapi (blue) and merged images. Scale bar: 20 μm. **c** The images (left) stand for caspase3 3D fluorescent staining and the bar graph (right) shows the ratio of caspase3 positive to total cells. Caspase3 (green), dapi (blue). Scale bar: 20 μm. Data are presented as the mean ± s.e.m. (*n* = 20); *p* = 0.098. (PDF 4348 kb)

Additional file 4: **Figure S4.** The path of single cell migration in control (left) and Col13-expressing MCF-10A cells (right); *n* = 13. (PDF 1546 kb)

Additional file 5: **Figure S5.** Transwell analysis of T4–2 cell invasion. **a** Quantification data showing invasion of control and Col13^−/−^ T4–2 cells. Data are presented as the mean ± s.e.m.; *n* = 3; * *p <* 0.05. **b** Quantification data showing invasion of control and Col13-expressing T4–2 cells. Data are presented as the mean ± s.e.m.; *n* = 3; n.s., no significance. (PDF 1423 kb)

Additional file 6: **Figure S6.** Co-culture invasion analysis in Transwell. Images (upper) and quantification data (lower) showing the invasion of GFP-labeled MDA-MB-231-vector control cells mixed with wild type MDA-MB-231 cells (1:1), GFP-labeled Col13^−/−^ MDA-MB-231 cells alone, and GFP-labeled Col13^−/−^ MDA-MB-231 cells mixed with wild type MDA-MB-231-Control cells (1:1). Each group had same amount of cells plated in the upper chamber; total cell number plated in the upper chamber is 0.1 M. The invaded GFP-labeled cell numbers were counted, and the number of GFP-labeled Col13^−/−^MDA-MB-231 alone group was divided by 2. Data are presented as the mean ± s.e.m. *n* = 3; ** *p <* 0.01. n.s., no significance. Scale bar: 50 μm. (PDF 4617 kb)

Additional file 7: **Figure S7.** Tumorsphere forming efficiency in T4–2 cells. **a** Phase images (left) and quantification data (right) showed tumorshpere formation efficiency in control and Col13^−/−^T4–2 cells. Data are presented as the mean ± s.e.m. *n* = 3; * *p* *<* 0.05. Scale bar: 100 μm. **b** Phase images (left) and quantification data (right) showed tumorshpere formation efficiency in control and Col13-expressing T4–2 cells. Data are presented as the mean ± s.e.m. *n* = 3; n.s., no significance. Scale bar: 100 μm. (PDF 5721 kb)

Additional file 8: **Figure S8.** Co-culture tumorsphere forming efficiency analysis. Quantification data showing tumorsphere forming efficiency of GFP-labeled MDA-MB-231 vector control cells mixed with wild type MDA-MB-231 cells (1:1), GFP-labeled Col13^−/−^ MDA-MB-231 cells alone, and GFP-labeled Col13^−/−^ MDA-MB-231 mixed with wild type MDA-MB-231 cells (1:1). Each group had the same amount of cells plated on poly-HEMA coated dishes, and GFP-labeled tumorsphere was counted, and the tumorsphere number of GFP-labeled Col13^−/−^ MDA-MB-231alone group was divided by 2. Data are presented as the mean ± s.e.m. *n* = 3; * *p* *<* 0.05, ** *p* *<* 0.01. n.s., no significance. (PDF 1452 kb)

Additional file 9: **Figure S9.** Kaplan-Meier analysis of distant recurrence free survival in ER positive breast cancer patients; the patients were equally divided into two groups based on the mRNA level of collagen XIII. *n* = 577. ***p < 0.001. (PDF 2351 kb)

Additional file 10: **Figure S10.** Western blot confirming Col13 knockout in 231-luc-D3H2LN cells. (PDF 1372 kb)

Additional file 11: **Figure S11.** Bright field and fluorescence images showing bone metastasis of GFP-labeled MDA-MB-231 cells in nude mice. Left two images showed bone metastasis of the control MAD-MB-231 cells on the hind leg after intracardiac inoculation. Right two images showed bone metastasis of Col13^−/−^ MAD-MB-231 cells on the fore leg after intracardiac inoculation. *n* = 4. (PDF 7411 kb)

## Abbreviations

CRISPR: Clustered regularly interspaced short palindromic repeats
DDRs: Discoidin domain receptors
ECM: Extracellular matrix
ER: Estrogen receptor
IVIS: in vivo imaging system
NC: Non-collagenous
SCID: Severe combined immun deficiency
TNBC: Triple-negative breast cancer
