# Supplementary figures and images for: Membrane associated collagen XIII promotes cancer metastasis and enhances anoikis resistance
Source: Breast Cancer Res. 2018 Oct 1;20:116. doi: 10.1186/s13058-018-1030-y (PMC6167877; doi:10.1186/s13058-018-1030-y)

Figure S1.

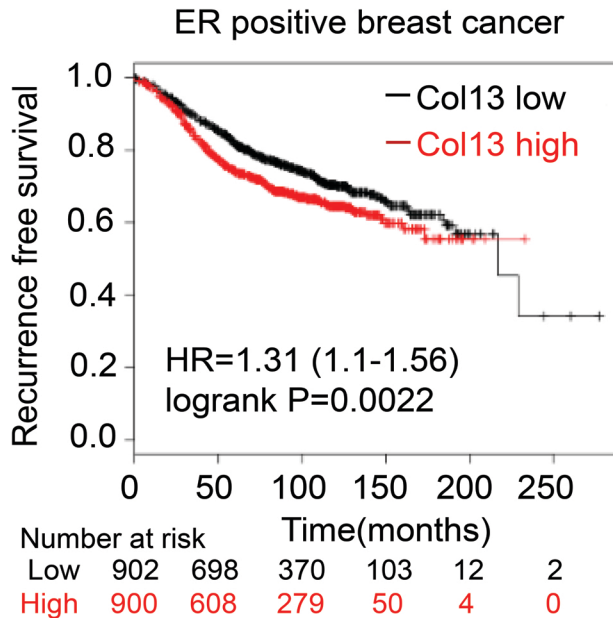

Supplement: Supplementary file 1 — Figure S1. Kaplan-Meier analysis of recurrence free survival in ER positive breast cancer patients; the patients were equally divided into two groups based on the mRNA level of Col13 in breast cancer tissue. n = 1802. **p < 0.01. (PDF 2061 kb) [file 13058_2018_1030_MOESM1_ESM.pdf]

Figure S2.

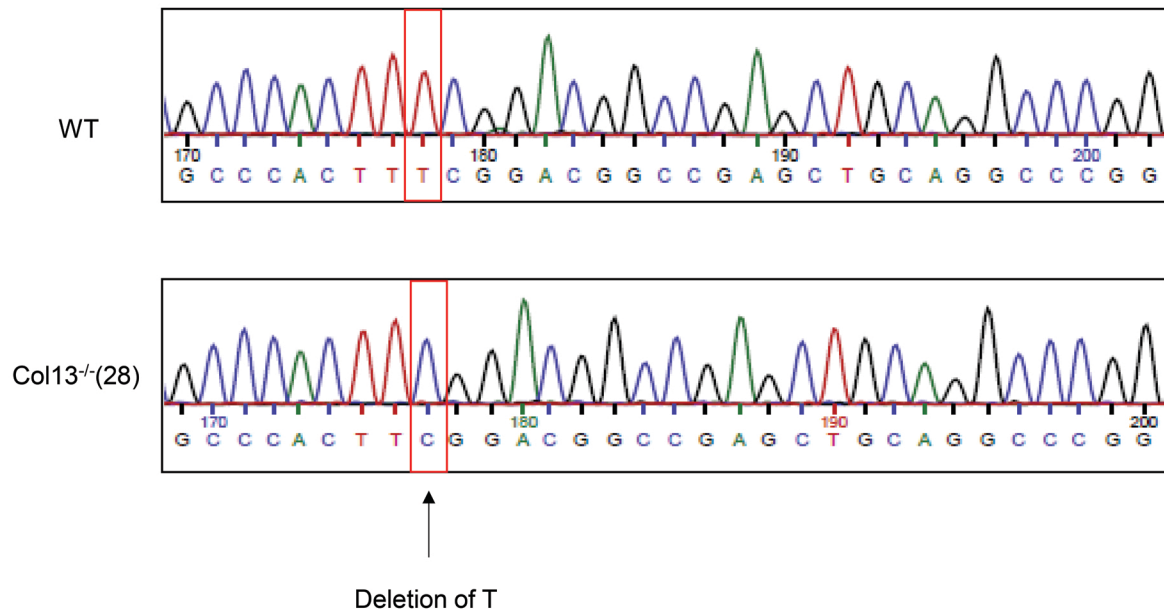

Supplement: Supplementary file 2 — Figure S2. Genomic DNA sequencing results of wild type and Col13 knockout MDA-MB-231 clone 28. There is a T deleted in the Col13 knockout MDA-MB-231 cells. (PDF 3096 kb) [file 13058_2018_1030_MOESM2_ESM.pdf]

Figure S3.

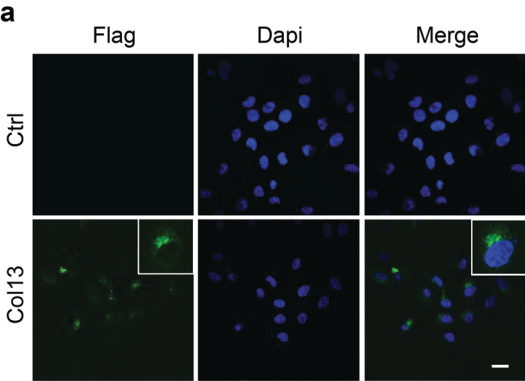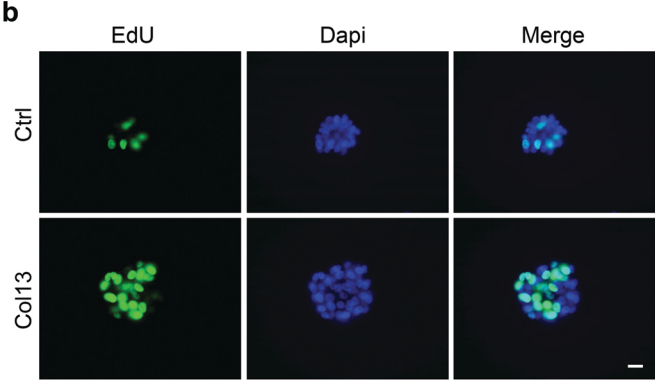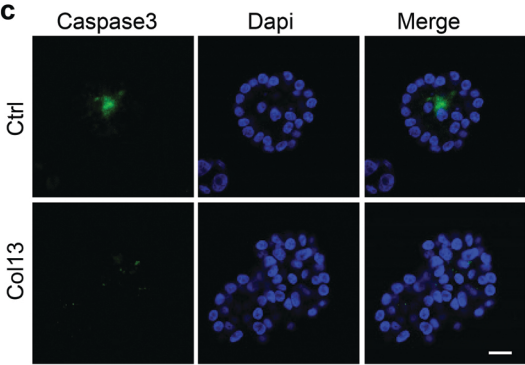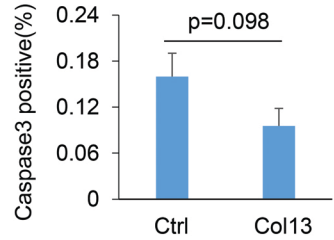

Supplement: Supplementary file 3 — Figure S3. Immunofluorescence staining analyzes collagen XIII expression, EdU labeling, and caspase 3 activation. a 2D culture fluorescent microscopy images of MCF-10A control and Col13 overexpression cells. Flag (green), dapi (blue) and merged images. Scale bar: 20 μm. b EdU staining fluorescent microscopy images of MCF-10A control and Col13 overexpression cells. Representative image was on day6 of 3D culture. EdU (green), dapi (blue) and merged images. Scale bar: 20 μm. c The images (left) stand for caspase3 3D fluorescent staining and the bar graph (right) shows the ratio of caspase3 positive to total cells. Caspase3 (green), dapi (blue). Scale bar: 20 μm. Data are presented as the mean ± s.e.m. (n = 20); p = 0.098. (PDF 4348 kb) [file 13058_2018_1030_MOESM3_ESM.pdf]

Figure S4.

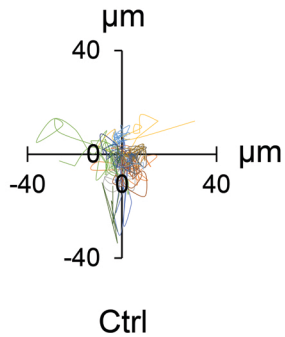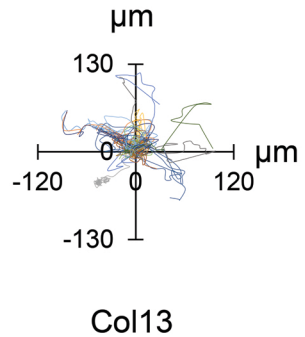

Supplement: Supplementary file 4 — Figure S4. The path of single cell migration in control (left) and Col13-expressing MCF-10A cells (right); n = 13. (PDF 1546 kb) [file 13058_2018_1030_MOESM4_ESM.pdf]

Figure S5.

**a**

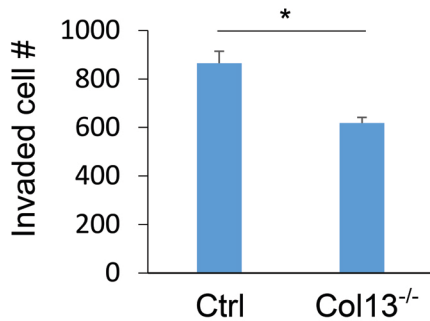

**b**

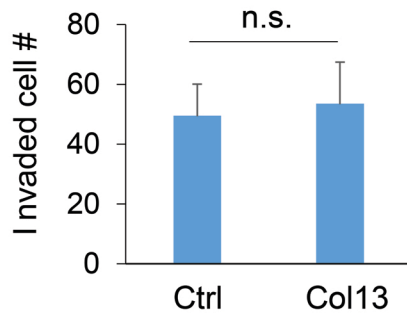

Supplement: Supplementary file 5 — Figure S5. Transwell analysis of T4–2 cell invasion. a Quantification data showing invasion of control and Col13−/− T4–2 cells. Data are presented as the mean ± s.e.m.; n = 3; * p < 0.05. b Quantification data showing invasion of control and Col13-expressing T4–2 cells. Data are presented as the mean ± s.e.m.; n = 3; n.s., no significance. (PDF 1423 kb) [file 13058_2018_1030_MOESM5_ESM.pdf]

Figure S6.

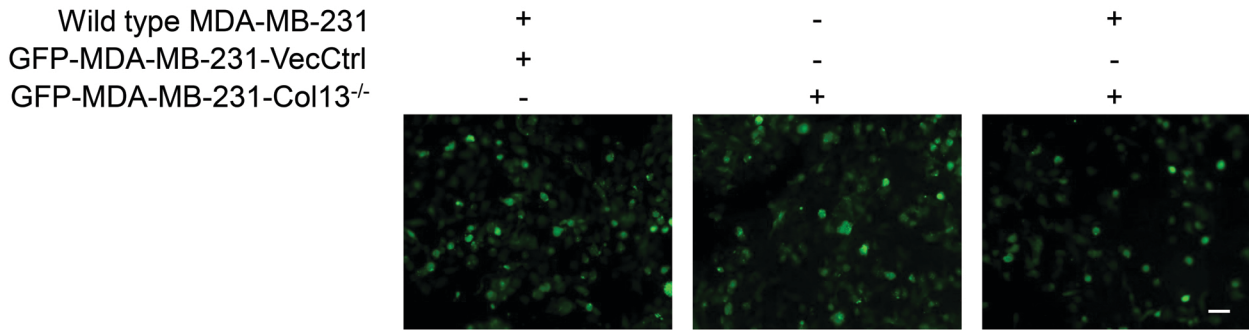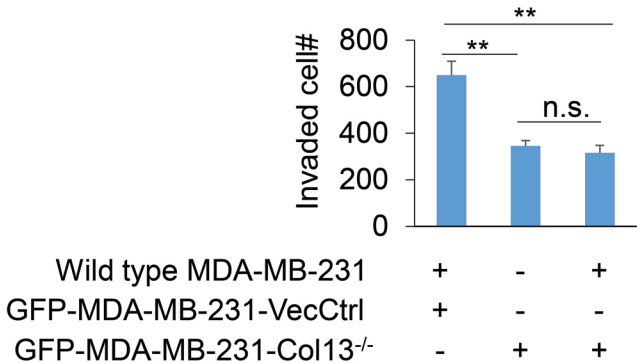

Supplement: Supplementary file 6 — Figure S6. Co-culture invasion analysis in Transwell. Images (upper) and quantification data (lower) showing the invasion of GFP-labeled MDA-MB-231-vector control cells mixed with wild type MDA-MB-231 cells (1:1), GFP-labeled Col13−/− MDA-MB-231 cells alone, and GFP-labeled Col13−/− MDA-MB-231 cells mixed with wild type MDA-MB-231-Control cells (1:1). Each group had same amount of cells plated in the upper chamber; total cell number plated in the upper chamber is 0.1 M. The invaded GFP-labeled cell numbers were counted, and the number of GFP-labeled Col13−/−MDA-MB-231 alone group was divided by 2. Data are presented as the mean ± s.e.m. n = 3; ** p < 0.01. n.s., no significance. Scale bar: 50 μm. (PDF 4617 kb) [file 13058_2018_1030_MOESM6_ESM.pdf]

Figure S7.

**a**

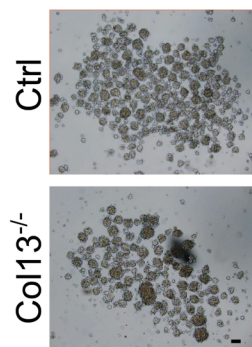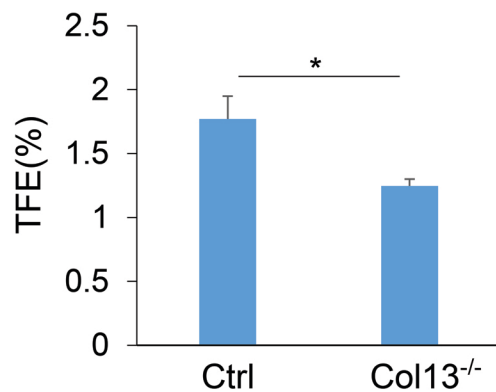

**b**

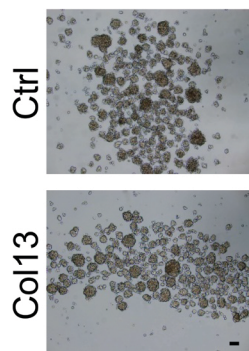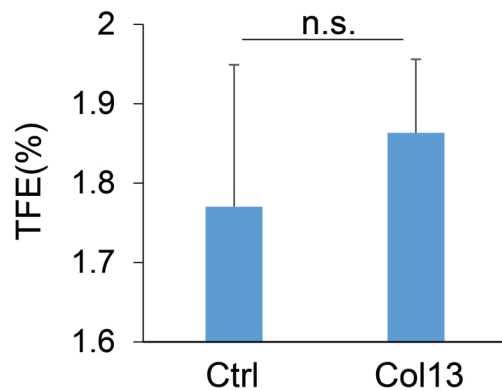

Supplement: Supplementary file 7 — Figure S7. Tumorsphere forming efficiency in T4–2 cells. a Phase images (left) and quantification data (right) showed tumorshpere formation efficiency in control and Col13−/−T4–2 cells. Data are presented as the mean ± s.e.m. n = 3; * p < 0.05. Scale bar: 100 μm. b Phase images (left) and quantification data (right) showed tumorshpere formation efficiency in control and Col13-expressing T4–2 cells. Data are presented as the mean ± s.e.m. n = 3; n.s., no significance. Scale bar: 100 μm. (PDF 5721 kb) [file 13058_2018_1030_MOESM7_ESM.pdf]

Figure S8.

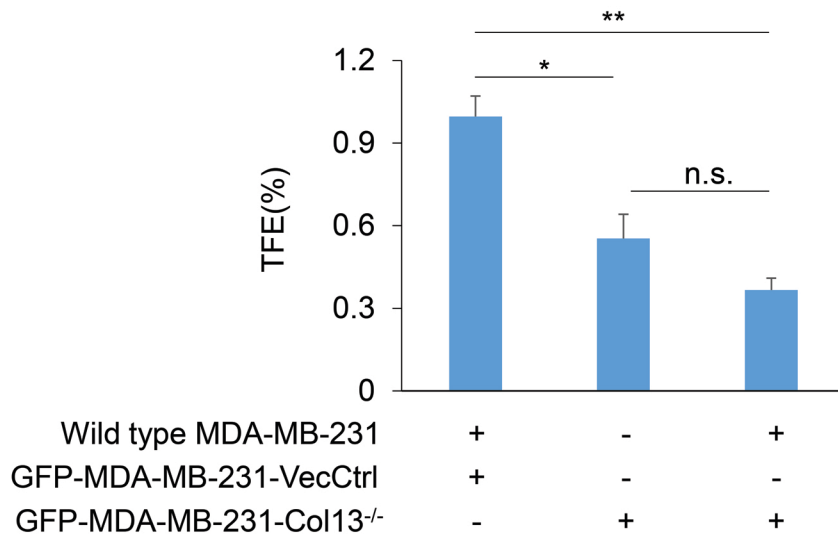

Supplement: Supplementary file 8 — Figure S8. Co-culture tumorsphere forming efficiency analysis. Quantification data showing tumorsphere forming efficiency of GFP-labeled MDA-MB-231 vector control cells mixed with wild type MDA-MB-231 cells (1:1), GFP-labeled Col13−/− MDA-MB-231 cells alone, and GFP-labeled Col13−/− MDA-MB-231 mixed with wild type MDA-MB-231 cells (1:1). Each group had the same amount of cells plated on poly-HEMA coated dishes, and GFP-labeled tumorsphere was counted, and the tumorsphere number of GFP-labeled Col13−/− MDA-MB-231alone group was divided by 2. Data are presented as the mean ± s.e.m. n = 3; * p < 0.05, ** p < 0.01. n.s., no significance. (PDF 1452 kb) [file 13058_2018_1030_MOESM8_ESM.pdf]

Figure S9.

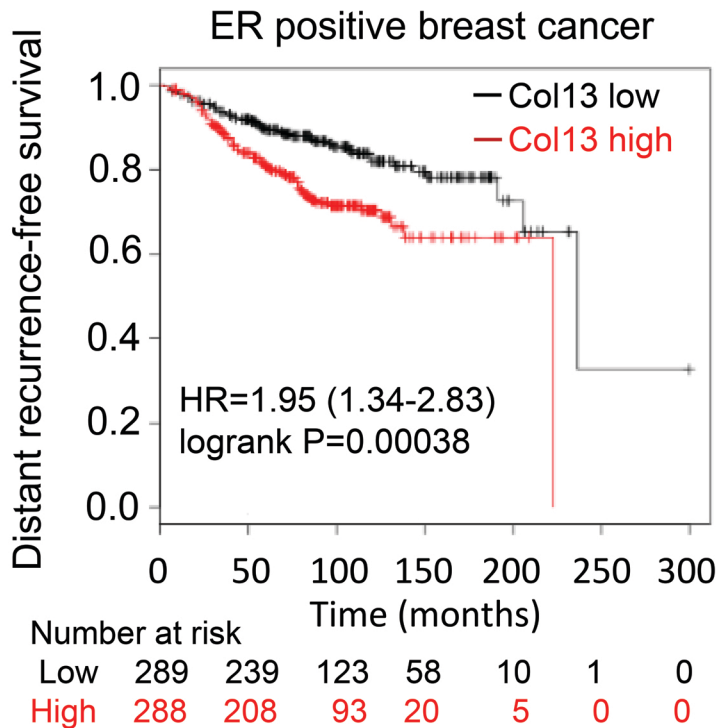

Supplement: Supplementary file 9 — Figure S9. Kaplan-Meier analysis of distant recurrence free survival in ER positive breast cancer patients; the patients were equally divided into two groups based on the mRNA level of collagen XIII. n = 577. ***p < 0.001. (PDF 2351 kb) [file 13058_2018_1030_MOESM9_ESM.pdf]

Figure S10.

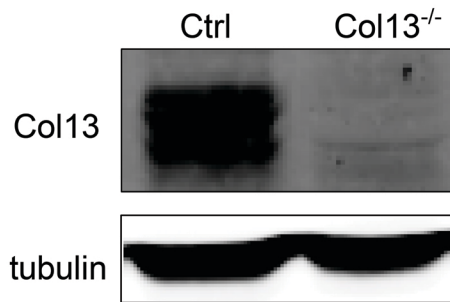

Supplement: Supplementary file 10 — Figure S10. Western blot confirming Col13 knockout in 231-luc-D3H2LN cells. (PDF 1372 kb) [file 13058_2018_1030_MOESM10_ESM.pdf]

Figure S11.

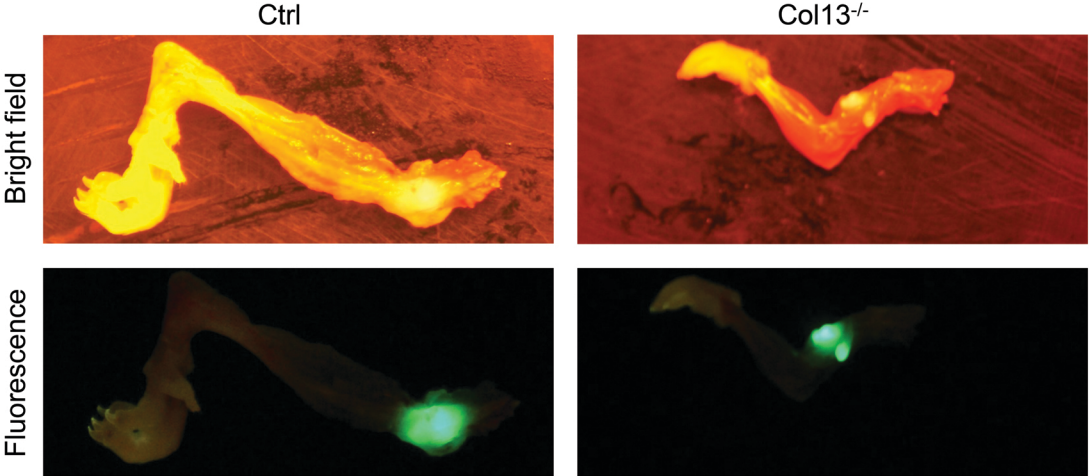

Supplement: Supplementary file 11 — Figure S11. Bright field and fluorescence images showing bone metastasis of GFP-labeled MDA-MB-231 cells in nude mice. Left two images showed bone metastasis of the control MAD-MB-231 cells on the hind leg after intracardiac inoculation. Right two images showed bone metastasis of Col13−/− MAD-MB-231 cells on the fore leg after intracardiac inoculation. n = 4. (PDF 7411 kb) [file 13058_2018_1030_MOESM11_ESM.pdf]
